# Physical Function Training Strategies for Improving Tennis Players’ Baseline Movement Stroke Ability

**DOI:** 10.1155/abb/7898511

**Published:** 2025-10-29

**Authors:** Lin Lu, Dan Wu, Yuanyuan Lei

**Affiliations:** ^1^ Department of Physical Education, Kunsan National University, Gunsan-Si, 541150, Republic of Korea, kunsan.ac.kr; ^2^ School of Physical Education, Hebei Normal University, Shijiazhuang, 050024, China, hebtu.edu.cn

**Keywords:** accuracy, comparative experiment, FMS test, physical function training, *t*-test, tennis stroke

## Abstract

**Objective:**

To investigate the effect of physical function training (PFT) on the improvement of tennis players’ baseline movement stroke ability and to verify the effectiveness of PFT.

**Method:**

Using a randomized controlled design, 32 national level 2 and above athletes (16 males and 16 females) from the tennis team of Chengdu Sport University were selected and randomly divided into an experimental group (EG; *n* = 16) and a control group (CG; *n* = 16). The EG received 12 weeks of physical training intervention, three times a week for 60 min each time. The CG underwent routine training on tennis balls. Before and after training, functional movement screens (FMSs), specialized physical fitness tests (including 100 m sprint run, standing long jump, fan run, backhand lateral movement, and forehand lateral movement), and baseline movement stroke effect tests (hitting frequency, hitting depth, and hitting accuracy) were conducted on both groups of athletes. The collected data were statistically analyzed using SPSS 26.0 and Microsoft Excel software. Paired samples *t*‐test and independent samples *t*‐test were used to compare the differences between the two groups of athletes and to determine significant differences between the data based on *p*‐values.

**Results:**

According to the FMS test findings, the EG’s athletes significantly improved their functional movement capacity on all test items following training (*p* < 0.05), but the CG did not significantly alter. In the specialized physical fitness test, the EG showed significant training effects in standing long jump, fan run, backhand lateral movement, and forehand lateral movement (*p* < 0.05). The CG displayed no significant training effect. The baseline movement stroke effect test showed that the EG athletes significantly increased the number of baseline forehand movement hitting, baseline backhand movement strokes, and baseline one forehand and one backhand movement strokes after training (*p* < 0.05). The depth and accuracy of the stroke also increased significantly (*p* < 0.05). However, although the number of strokes increased in the CG athletes, the improvement in depth of stroke and accuracy was not significant (*p* > 0.05).

**Conclusion:**

The 12‐week physical training intervention can significantly improve the functional movement quality, specialized movement efficiency, and baseline hitting performance of college tennis players. This provides evidence‐based training programs for improving baseline hitting ability.

## 1. Introduction

Tennis is one of the most popular sports in the world. With the popularization of tennis on campus and among the masses, more and more people have the opportunity to contact and participate in tennis training [1]. However, as tennis has rapidly developed, improving athletes’ competitive level and baseline stroke efficiency has become crucial for determining the outcome of points in modern rallies. Baseline movement stroke ability is an important indicator of the overall technical level of tennis players, which is directly related to the wins and losses of players in the game [2]. Efficient baseline strokes not only require athletes to master precise technical movements, but also require them to have good physical fitness and coordination. However, traditional tennis training methods either mechanically repeat shots or ignore real game situations. Reid et al.’s [3] research suggested that both approaches were difficult to truly optimize learning and long‐term performance, while more context changing exercises and modern skill acquisition methods that emphasize intrinsic feedback were more effective. Functional training (FT) is a training method that focuses on simulating the movement patterns of daily life and exercise. It emphasizes the participation of multiple joints and planes, as well as dynamic coordination and stability. Its goal was to enhance the overall functional efficiency of the neuromuscular system (such as strength, explosiveness, balance, flexibility, and coordination), improve individual performance, and injury prevention abilities in real activities, rather than solely pursuing isolated hypertrophy or maximum strength of a muscle. Training often combines unstable planes, free weights, compound movements, and task‐oriented loads to activate and optimize neuromuscular control, proprioception, and core stability. This achieves the transfer and practical application of “functional strength” [4].

Yahya et al. [5] found that physical function training (PFT) can prevent sports injuries by developing the strength of deep small muscles and improve athletes’ motor coordination, thus, improving athletes’ competitive level in general. Tokhirzhonovich [6] analyzed the effect of PFT on athletes’ physical training. The results revealed that the effect of body movement FT was significant and safe and reliable, the training place was flexible, the equipment was simple, and the methods were diverse. Through physical motor FT, it could effectively improve athletes’ specialized sports ability, reduce the occurrence of sports injuries, and promote physical recovery [6]. Valenzuela et al. [7] analyzed the concepts and characteristics of PFT theory and explored the feasibility of its integration into college physical education curriculum. The results showed that PFT theory was suitable for integration into college physical education programs [7].

Although physical training is widely recognized as a way to improve athletes’ competitiveness and fitness level, existing research on the relationship between general physical training and tennis performance is still relatively generalized. There has been little empirical analysis of FT interventions specifically designed for biomechanics. Therefore, this study innovatively designs and implements a 12‐week FT program targeting biomechanical characteristics. The study systematically examines the improvement effect of PFT on the baseline hitting efficiency of university tennis players through multidimensional evaluations such as functional movement screens (FMSs) testing, physical fitness testing, and baseline hitting effect testing. The aim is to verify the effectiveness and superiority of the proposed training program.

## 2. Information and Methods

### 2.1. General Information

The study was carried out at Chengdu Institute of Physical Education from June 1 to August 30, 2023, to look at the impact of baseline stoke and PFT on tennis players’ physical fitness. 32 national level 2 and above tennis players from the school’s tennis team were selected as the research subjects. Considering the significant gender differences in neuromuscular control and training responsiveness, the “gender stratification, block randomization” method was used for grouping. First, the 32 participants were divided into two groups based on gender: male and female. Each group used block randomization with a block length of four and generated a random sequence with SAS 9.4. This ensured that two out of every four participants of the same gender entered the experimental group (EG) and two enter the control group (CG). The final EG and CG consisted of 16 individuals each, with a male to female ratio of 1:1 (8 males and 8 females) in each group. Randomization was performed by independent statisticians, and participants in the experiment were blinded until the grouping was announced. Athletes in the EG underwent a 12‐week tennis training session with the physical motor FT intervention, while athletes in the CG underwent a regular tennis physical training session in the same environment and conditions. The inclusion criteria of the athletes were as follows: (1) Age between 20 and 22 years. (2) Athletes at Level 2 and above. (3) With more skillful mastery of tennis. (4) Body mass index within normal range. The exclusion criteria were as follows: (1) Poor obedience and lack of active cooperation in completing the experiment. (2) The presence of recent physical injuries. The college’s sports committee management board approved the entire experiment for the study, and the participating students completed an informed consent form.

The experimental site is located on the school’s tennis court and power room, and the tools used include the functional motion screening professional kit, a calibration ruler with a scale accuracy of 1 mm, used to test shoulder flexibility, MX T40 camera, used to capture athlete movements, infrared photoelectric timer with an accuracy of ±0.01 s, starting block, front pedal 50°, rear pedal 65°, a distance measuring ruler with an accuracy of ±0.1 cm, tennis ball (diameter ~6.54 cm, weight ~57.0 g), logo bucket (~35 cm in height, 15 cm in diameter, and available in red, yellow, and blue colors), dumbbells (weighing 5 pounds, 10 pounds, 20 pounds, 30 pounds, and 50 pounds), elastic bands (~4 m in length), yoga mats (~2.5 m in length, 0.8 m in width, and 10 cm in thickness), Swiss balls (with diameters of ~55 and 65 cm), solid balls (2 pounds, 4 pounds, 6 pounds, etc.), medicine balls (2 pounds, 4 pounds, 6 pounds, etc.), and polar team pro heart rate monitor [8, 9].

### 2.2. Research Methods

**Figure 1:**
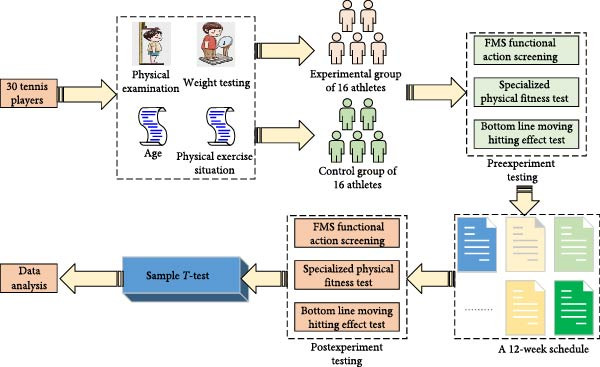
Flow chart of the experiment.

Prior to the commencement of the experiment, the height, weight, age, and physical activity of the participating athletes are routinely tested and investigated and accordingly they are divided into two groups. The EG receives a PFT program developed by the study, while the CG undergo regular tennis physical training sessions. To minimize evaluation bias, the evaluators responsible for conducting the posttest were unaware of the allocation of participants into groups. After a 12‐week training cycle, the study assesses and compares the differences in functional movements, physical fitness, and stroke performance between the athletes in the EG and the CG, so as to verify the effectiveness of PFT [10]. The specific experimental procedure is shown in Figure 1.

**Figure 2:**
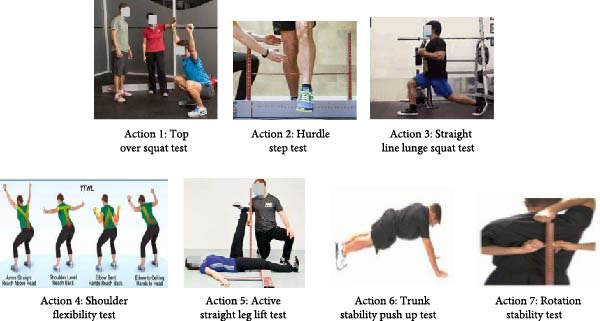
Graphical representation of FMS test items.

In Figure 1, before the beginning of the experiment, the tennis players in the EG and the CG are measured the indexes before the experiment, including three major items: FMS, specialized physical fitness test, and baseline movement stroke effect test. The FMS test program includes seven movements. The FMS test program includes seven movements that involve coordinated movements of multiple joints and muscle groups, such as over‐the‐squat and hurdle tests. These movements require the body to maintain proper joint alignment and muscle activation patterns in multiplane movements, reflecting the biomechanical efficiency of the human body in complex movement tasks. Second, the FMS testing program focuses on core stability and balance skills. These include rotational stability tests and core stability push‐ups. These tests challenge the body’s center of gravity control and the stability of the core muscle groups. They are used to evaluate the coordination and movement control abilities of the neuromuscular system. In addition, shoulder range of motion tests and active straight leg lift (ASLL) tests focus on evaluating joint range of motion and muscle flexibility to identify potential movement limitations, as shown in Figure 2.

As shown in Figure 2, FMS screening includes seven tests. Test one is the assessment of bilateral ankle, knee, and hip joint mobility as well as chest extension during squats. Then, the followed test is the assessment of gait mechanics and stability during single leg standing in hurdle steps. Linear bow and arrow step squat test is used to evaluate hip joint lateral control and stability when standing in a split position. Shoulder flexibility test evaluates the range of motion of both shoulders, including internal rotation and adduction. The ASLL test evaluates the flexibility of the hamstring and calf muscles while maintaining core stability. Trunk stability push‐up test evaluates core stability and spinal stiffness during closed chain upper‐body exercise. Rotational stability testing evaluates the stability and coordination of multiplanar cores during contralateral limb movement. A subject who successfully completes the required movement receives a score of 4, which is the highest possible score on the FMS exam. A subject who can perform the necessary movement in a consistent way receives a score of 3. A subject that successfully completes the exercise, but exhibits compensatory phenomena or instability receives a score of 2. If a subject is unable to finish the entire movement, they receive a score of 1. If the respondent felt discomfort during the exam, they would receive zero points [11–13].

**Figure 3:**
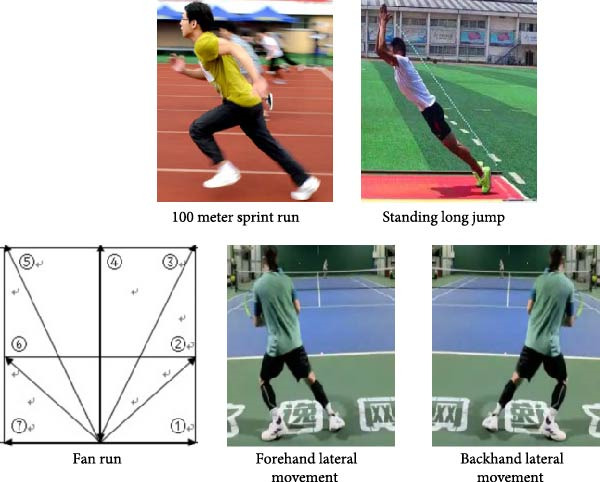
Specialized physical fitness test items.

The specialized physical fitness test includes five tests: a 100‐m sprint, a standing long jump, a fan‐shaped run, a lateral movement test (forehand and backhand), and a basic movement hitting test. These test items provide a scientific basis for the evaluation of athletes’ specialized skills through various movement modes and biomechanical mechanisms, which are shown in Figure 3.

In Figure 3, the 100‐m sprint is the gold standard on‐site test for evaluating line speed. Due to its high test–retest reliability and standard validity, it is widely used to measure acceleration and maximum operating speed.

The standing long jump is a fully validated test used to evaluate the generation of explosive and horizontal forces in the lower body.

In the fan run, participants quickly move along a designated path within a fan‐shaped area, and the duration is measured. These agility tests have the same kinematic characteristics as established tests and require rapid directional changes in set modes. These tests are recognized for evaluating agility, body control, and leg speed.

The forehand lateral movement test is a test of a tennis player’s lateral movement ability. The subjects were asked to start from one side of the baseline, move to the other side, simulate lateral movement in a tennis match, and record the time taken. Backhand lateral movement is similar to forehand lateral movement, but in the opposite direction. The subjects were asked to start from the other side of the baseline and move to the starting side. These tests simulate the defensive movements required for specific movements in tennis, testing the efficiency and agility of athletes moving in different directions.

The bottom line movement hitting effect test includes two indicators: hitting depth test and hitting accuracy test [14–16], which can evaluate the depth, accuracy, and strength of an athlete’s hitting at the bottom line. The testing area for the bottom line movement hitting effect is shown in Figure 4.

Figure 4Test site for baseline movement stroke effect. (a) Ground setting diagram for hitting depth. (b) Ground setting diagram for hitting accuracy.

**Figure:**
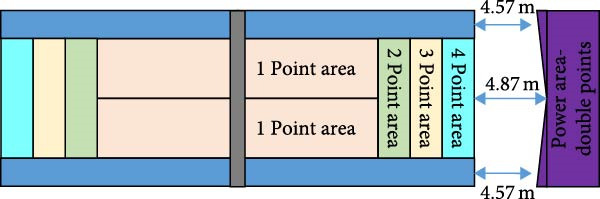
(a)

**Figure:**
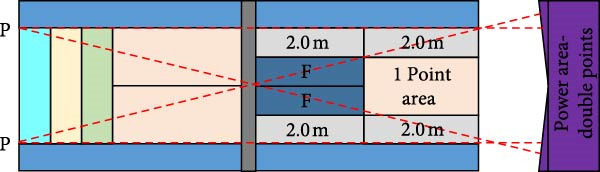
(b)

Figure 4a shows the tennis hitting depth court setup for hitting depth testing. The player is positioned in the center behind the baseline and takes turns hitting 10 balls with his or her forehand and backhand. The feeder is located at the midpoint of the intersection of the serve line and the net on the opposite side of the court to ensure that the ball flies to the forehand or backhand area of the player. The court is divided into four zones and scoring is based on the zone where the return ball lands [17]. Figure 4b shows the tennis stroke accuracy court setup for stroke accuracy testing [18]. The player performs 12 strokes. Among them, six strokes are alternating forehand and backhand straight line strokes and six strokes are alternating forehand and backhand diagonal strokes. The athlete stands in the center of the baseline and the feeder is at the midpoint of the serving line with the goal of maintaining the depth and angle of the ball. The court is marked with a line 2.0 m inward along the singles sideline, delineating four specific zones, and scoring is based on the drop zone. In addition, the athlete will be awarded one point for each successful stroke with no errors during the test. An additional 1 point is also added for moving and completing the stroke times on the baseline within the allotted time [19].

After completing the preexperimental pretest, the tennis players in the EG and CG start a 12‐week training program. The training is conducted three times a week for 1 h at a time, as shown in Table 1.

**Table 1:** 12‐week training schedule.

Week number	Physical function training/routine training	Time	Tennis technique training	Time	Physical function training/routine training	Time
1	Monday	15:00–16:00	Tuesday	16:00–17:00	Wednesday	15:00–16:00
2	Thursday	15:00–16:00	Friday	16:00–17:00	Saturday	15:00–16:00
3	Monday	15:00–16:00	Tuesday	16:00–17:00	Wednesday	15:00–16:00
4	Thursday	15:00–16:00	Friday	16:00–17:00	Saturday	15:00–16:00
5	Monday	15:00–16:00	Tuesday	16:00–17:00	Wednesday	15:00–16:00
6	Thursday	15:00–16:00	Friday	16:00–17:00	Saturday	15:00–16:00
7	Monday	15:00–16:00	Tuesday	16:00–17:00	Wednesday	15:00–16:00
8	Thursday	15:00–16:00	Friday	16:00–17:00	Saturday	15:00–16:00
9	Monday	15:00–16:00	Tuesday	16:00–17:00	Wednesday	15:00–16:00
10	Thursday	15:00–16:00	Friday	16:00–17:00	Saturday	15:00–16:00
11	Monday	15:00–16:00	Tuesday	16:00–17:00	Wednesday	15:00–16:00
12	Thursday	15:00–16:00	Friday	16:00–17:00	Saturday	15:00–16:00

According to the above schedule, the EG of athletes performs the PFT developed in the study. The CG of athletes performs the regular tennis physical training.

The EG athletes are trained as follows: (A) Muscle activation training: (1) Two sets of horizontal tension elastic bands for both arms for a total of 30 times. (2) Elbow flexion and extension two sets of 30 times. (3) Single‐leg exchange elastic band pulling two groups totaling 30 times. (B) Stretching and warm‐up exercises: (1) One set of dynamic stretching two times. (2) Static stretching one set of two times. (3) Stretching hip flexors one group of 10 times. (4) Single leg support folding stretch one group of 10 times. (C) Upper‐body training: (1) Single foot elevated push‐ups two sets of 20 reps. (2) Double‐leg elevated push‐ups one set of 10 reps. (3) Stretch rope high and low rotations two sets totaling 10 reps. (4) Two sets of 2.5 kg solid ball chest thrusts for a total of 12 reps. (5) 2.5 kg solid ball one‐handed throw five sets totaling 10 times. (6) 2.5 kg medicine ball deep push‐up five sets of 10 reps. (D) Lower extremity training: (1) Rear foot elevation split‐leg squat two sets of 10 reps. (2) Double‐leg box jumps two sets of 10 times. (3) Swiss ball leg bends one group total 20 times. (4) Elastic band loaded single leg hard pull three sets of 18 reps. (5) Mini elastic band walking between rows one set of one time. (E) Trunk training: (1) Back bridge ine set of 10 reps. (2) Single‐leg side bridge one set of 10 reps. (3) Side bridge swinging arm turn one set of one time. (4) Supine abdominal turn two sets of 10 times. (5) Single knee kneeling support balance plate side throw solid ball two sets of 16 times. (F) Stretching and recovery exercises: (1) Foam axle rolling trunk piriformis two sets of 20 reps. (2) Foam axle rolling latissimus dorsi for two sets of 20 reps. (3) Static side leg press (both sides) one set totaling one rep. (4) Static lunge leg press (both legs) one set totaling one rep. (5) Brettzel twist pulls one set totaling 10 reps.

In the training of the EG athletes, six training contents are comprehensively applied to the muscles, bones, joints, and neural control system of the human body through different biomechanical principles. Therefore, it significantly improves the athletes’ physical function and performance. Muscle activation training (A) simulates the coordinated mechanisms of shoulder strap stability and lower‐limb support during tennis forehand and backhand strokes. MAT uses elastic band lateral tension and single‐leg stability movements to enhance scapular stability and lower‐limb neuromuscular control in dynamic orientations. The stretching and warm‐up routine (B) focuses on enhancing the range of motion of the hip joints and flexibility of the trunk rotation through a combination of dynamic and static methods. This routine is closely related to the maximum range of motion of the joints and muscle elasticity required for a tennis serve or interception. Upper‐limb training (C) emphasizes pushing, throwing, and rotating movements. These movements include solid ball chest pushing and one‐handed throwing. They directly correspond to the power output and momentum transfer mechanism of the chest‐shoulder chain during serves and high‐pressure hits. Lower‐limb training (D) improves the efficiency of lower limb extension and landing buffering ability. This training involves exercises such as leg splitting squats and jumping boxes. These exercises conform to the biomechanical characteristics of frequent jumps, emergency stops, and lateral kicks in tennis. Trunk training (E) focuses primarily on bridge, spin, and side throw movements. These movements strengthen the core’s ability to generate and transmit torque during the hitting process. This is key to efficient power generation and reducing energy leakage. Finally, the recovery training (F) counteracts the tension in the thoracic vertebrae, piriformis, and latissimus dorsi that is commonly experienced by tennis players. This is achieved through foam axis relaxation and static stretching, which maintain muscle elasticity and prevent compensatory injuries caused by repeated rotation and swing. The entire plan systematically optimizes the power chain efficiency, stability, and recovery ability required for tennis from a biomechanical perspective.

The training content of the CG is as follows: (A) Muscle activation training: (1) Foam axle rolling upper back one group totaling 10 times. (2) Foam axis rolling calves two groups totaling 20 times. (3) Foam axis rolling thighs front and back two groups of 20 times. (4) One set of horizontal stretching elastic band for both arms for 10 times. (B) Stretching and warm‐up exercises: (1) Static stretching one set of two reps. (2) Static stretching one set of two times. (C) Upper‐body training: (1) Dumbbell overhead press one set of 10 reps. (2) Dumbbell elbow flexion extension one group of 10 times. (3) Throw a solid ball two sets of 20 times. (4) Dumbbell swing one set of 20 reps. (5) Dumbbell double arm lateral raise two sets of 10 reps. (D) Lower‐body training: (1) Barbell squats two sets of 20 reps. (2) Kneeling abdominal wheel two sets of 10 times. (3) Side plate support two sets of 10 times. (4) Squat jumps two sets of 20 times. (5) Single‐leg pull‐ups three sets of six reps. (6) Kneeling squat two sets of 10 reps. (E) Torso training: (1) Abdominal breathing two sets of 10 times. (2) Plank support two sets of 10 reps. (3) Hip bridge one set of one time. (4) Lying posture touching the toes two groups total of 20 times. (5) Two groups of 30 times. (6) Single leg hip bridge (30 s) three sets. (F) Stretching and recovery exercises: (1) Static stretching one set of two reps. (2) Foam axis rolling upper back one group total 10 times. (3) Foam axle rolling calf two sets of 10 times. (4) Foam axis rolling anterior and posterior thighs one set of 10 times [20, 21].

At the conclusion of a 12‐week training period, all subject athletes take the experimental posttest. The test items and rubrics are consistent with the experimental pretest.

### 2.3. Methods of Statistical Analysis of Data

During the pretest and posttest of the experiment, the athletes in the EG and CG has their experimental data promptly collected. The collected data are then statistically analyzed using SPSS 26.0 and Microsoft Excel software. Before selecting the parameter testing method, the study first uses the Shapiro–Wilk test to verify the normality of the data. For data that follows a normal distribution, paired sample *t*‐test is used for intragroup comparison before and after, and independent sample *t*‐test is used for intergroup comparison [22]. The results of the experiments are presented in the format of “mean ± standard deviation” and are analyzed mathematically using Stata 12.0 software. *p* < 0.05 indicates a statistically significant difference (SSD) [23]. To compensate for the limitations of small sample sizes in interpreting results, the effect size of each key movement indicator is calculated. For intergroup comparisons (EG posttest and CG posttest), Cohen’s *d* is used. Moreover, for intragroup comparisons (EG pretest and posttest), Cohen’s *d*_*z*_ is used. Then 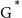
Power software is used to calculate statistical power. Effect size (*d*/*d*_*z*_): |*d*| < 0.2 (negligible), 0.2 ≤ |*d*| < 0.5 (small), 0.5 ≤ |*d*| < 0.8 (medium), |*d*| ≥ 0.8 (large). Statistical power: ≥0.8 (80%) indicates sufficient sample size. If it is less than 0.8, it is considered that the sample size is insufficient and there is a risk of Class II errors.

## 3. Result

### 3.1. Baseline Data Analysis

The statistical results of the baseline information of the patients in the EG and CG are shown in Table 2. All continuous variables were confirmed to follow a normal distribution by Shapiro–Wilk test (*p* > 0.05), and intergroup homogeneity of variance was confirmed by Levene’s test (*p* > 0.05), meeting the conditions for parameter testing. The independent sample *t*‐test results showed no significant differences between the two groups in terms of age, average gender, average weight, routine blood tests, and other indicators (*p* > 0.05), thus, excluding the interference of these factors on the results of the study. These subject athletes did not have significant variability in their physical parameters, thus, allowing for subsequent experiments.

**Table 2:** Statistics of patients’ baseline information.

Project		Experimental group	Control group	*p*‐Value	*t*‐Value
Sex ratio		1:1	1:1	*p* > 0.05	1.32
Average age (years)		21.36 ± 1.02	21.24 ± 1.12	*p* > 0.05	1.02
Average weight of male patients (kg)		65.63 ± 4.12	66.12 ± 3.65	*p* > 0.05	0.96
Average weight of female patients (kg)		49.82 ± 4.24	50.12 ± 3.45	*p* > 0.05	1.21
Body fat percentage (%)		23.23 ± 4.21	23.12 ± 4.09	*p* > 0.05	1.36
Muscle percentage (%)		37.12 ± 3.67	37.06 ± 3.71	*p* > 0.05	1.58
Blood routine	All normal		All normal		
Renal function	All normal		All normal		
Past medical history	No		No		

### 3.2. FMS Test Program

The FMS test items included top over squat, hurdle step, straight line lunge squat, shoulder flexibility, the ASLL test, trunk stability push up, rotation, and stability. Seven body movements were tested and represented as “1,”, “2,”, “3,”, “4,”, “5,”, “6,”, and “7,” respectively. The study compiled and analyzed the data of FMS test situation before and after training of both groups of athletes. The results are shown in Figure 5.

Figure 5Comparison of FMS test before and after training in two groups of athletes. (a) Control group. (b) Experimental group.

**Figure:**
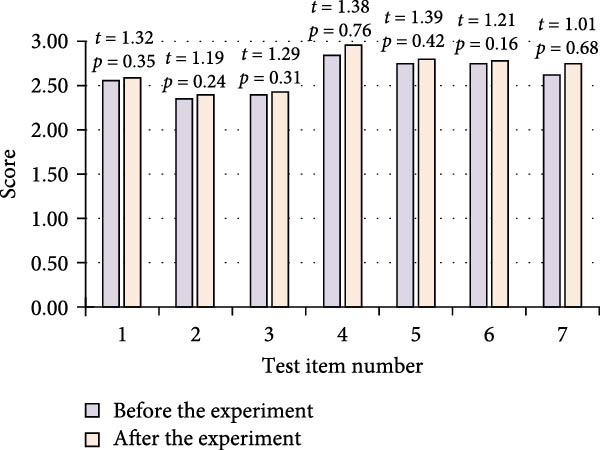
(a)

**Figure:**
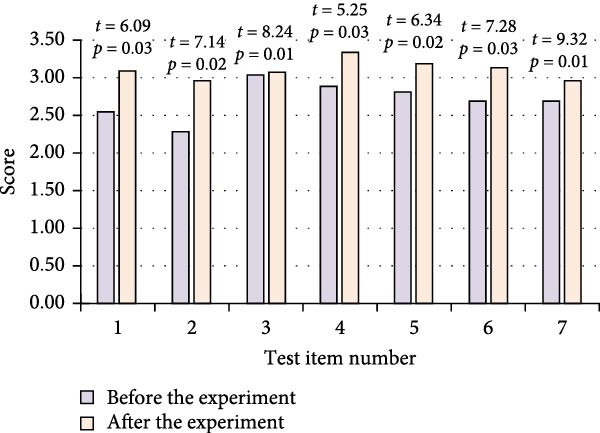
(b)

In Figure 5, in the CG, the *p*‐value of each test item before and after the training of the athletes was greater than 0.05. Although there was a slight improvement in some of the items such as shoulder flexibility from 2.84 ± 0.02 to 2.96 ± 0.03 and the ASLL test from 2.76 ± 0.03 to 2.81 ± 0.03, the overall improvement was not significant. However, for the EG of athletes, the *p*‐values were less than 0.05 after training. This demonstrated that these athletes’ functional movement skills significantly improved on all test items following training.

The FMS test included seven action items, with a total score range of 0–21 points. All score data conformed to a normal distribution after passing the Shapiro–Wilk test. The composite total score statistics showed that the EG had a composite total score of 14.2 ± 1.8 points before training, while the CG had a composite total score of 14.5 ± 1.6 points, and each athlete’s score is greater than 14. After training, the EG significantly improved to 17.8 ± 1.5 points, an increase of 25.4% compared to before training. The CG scored 15.1 ± 1.7 points, an increase of 4.1% compared to before training. Further demonstrated that the intervention measures of the EG had a specific effect on improving the total score of FMS.

### 3.3. Analysis of Results of Specialized Physical Fitness Tests

Specialized physical fitness test included 100‐m sprint run, standing long jump, fan run, backhand lateral movement, forehand lateral movement five events. They were numbered with “a,” “b,” “c,” “d,” and “e,” respectively. Each item was tested three times. For example, For example, “a(1)” represents the average value of the first test of the 100 m sprint run. “e(2)” represents the average value of the second test of forehand lateral movement. The results of the 100‐m sprint run test are shown in Figure 6.

Figure 6Comparison of 100 m sprint run results before and after training for both groups of athletes. (a) Control group. (b) Experimental group.

**Figure:**
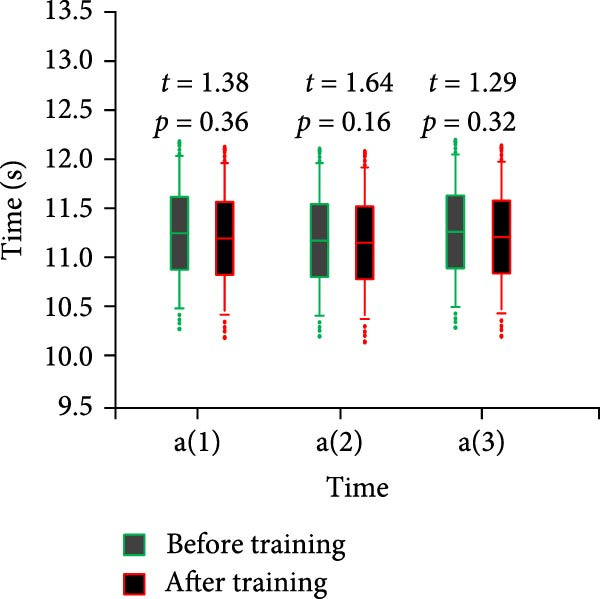
(a)

**Figure:**
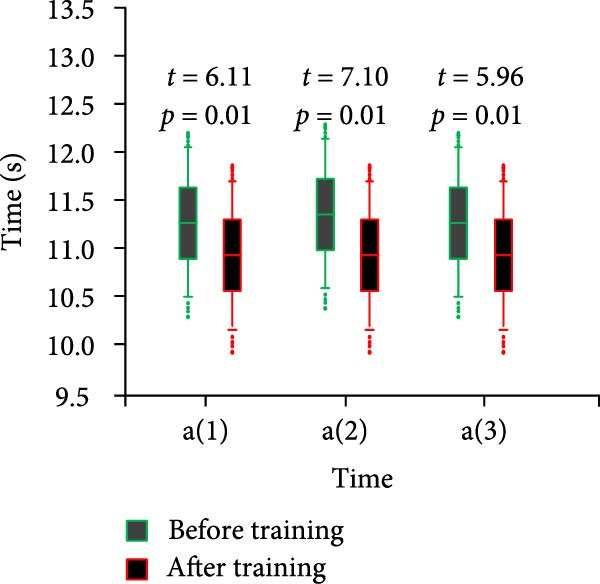
(b)

In Figure 6a, the mean time of the three tests of 100‐m sprint run before training of the CG athletes was 11.25 ± 0.24. The mean time of the posttraining period was 11.24 ± 0.31 and *p* < 0.05. In Figure 6b, the mean time of the three tests of 100‐m sprint run before training for the control athletes was 11.24 ± 0.21. The mean time after training was 10.99 ± 0.21 and *p* < 0.05. Table 3 displays the standing long jump and fan run test results.

**Table 3:** Comparison of test results of standing long jump and fan run before and after training of two groups of athletes.

Group	Number of tests	Before training	After training	*t*‐Values	*p*‐Values
Control group	b(1)	2.53 ± 0.23	2.55 ± 0.19	1.63	0.15
	b(2)	2.52 ± 0.21	2.54 ± 0.23	1.86	0.63
	b(3)	2.52 ± 0.19	2.55 ± 0.22	1.96	0.54

Experimental group	b(1)	2.51 ± 0.23	2.58 ± 0.32	7.63	<0.05
	b(2)	2.53 ± 0.27	2.59 ± 0.19	6.35	<0.05
	b(3)	2.52 ± 0.26	2.60 ± 0.23	6.96	<0.05

Control group	c(1)	16.32 ± 0.11	16.63 ± 0.21	1.85	0.63
	c(2)	16.65 ± 0.21	16.56 ± 0.14	1.76	0.45
	c(3)	16.74 ± 0.25	16.64 ± 0.32	1.35	0.53

Experimental group	c(1)	16.57 ± 0.24	17.76 ± 0.19	9.63	<0.05
	c(2)	16.61 ± 0.23	17.69 ± 0.23	7.63	<0.05
	c(3)	16.54 ± 0.37	17.74 ± 0.24	8.54	<0.05

In Table 3, on the standing long jump, although the CG displayed a slight increase in the mean after training, the *t*‐values indicated that this increase was not significant. Additionally, all of the *p*‐values were higher than 0.05, indicating that there was no significant difference between pre‐ and posttraining. The CG’s training effect on the fan run was likewise not significant, as evidenced by low *t*‐values and *p*‐values that were all higher than 0.05. In contrast, the EG showed significant training effects on both the standing long jump and the fan run. The significant enhancement of the mean values after training with high *t*‐values and *p*‐values less than 0.05 indicate that this enhancement was statistically significant. In conclusion, while there was no discernible training benefit in the CG, the training intervention in the EG significantly improved standing long jump and fan run performance. The test outcomes of backhand lateral movement and forehand lateral movement are shown in Figure 7.

Figure 7Test results of backhand lateral movement and forehand lateral movement before and after training for both groups of athletes. (a) Forehand lateral movement. (b) Backhand lateral movement.

**Figure:**
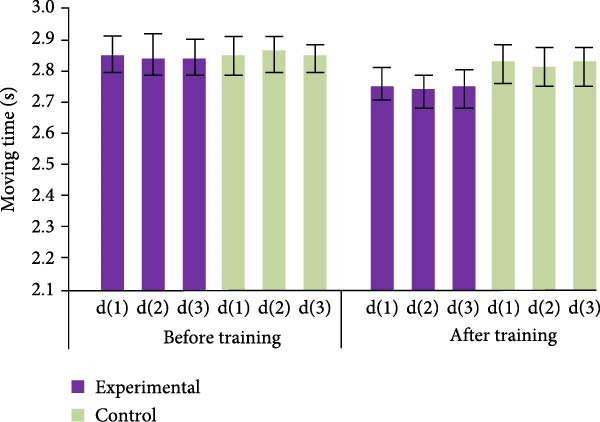
(a)

**Figure:**
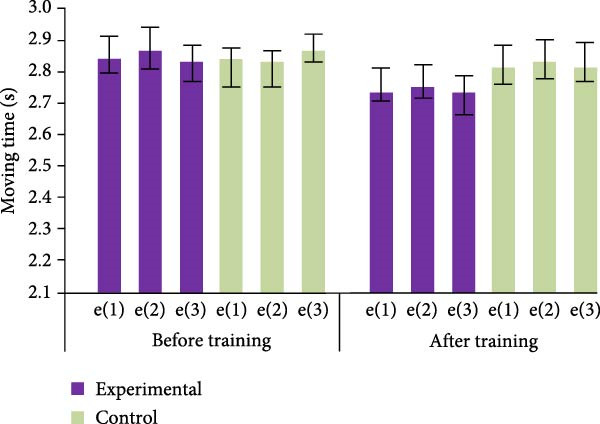
(b)

In Figure 7a, the EG athletes showed a significant decrease in the duration of forehand lateral movement after undergoing PFT, from an average duration of approximately 2.86 ± 0.53 to 2.76 ± 0.63 s. The CG athletes reduced their average time from about 2.85 ± 0.49 to 2.82 ± 0.51 s, but the effect was not significant. Similarly, in Figure 7b, after training, the average time for backhand lateral movement of the EG athletes decreased from 2.82 ± 0.35 to 2.73 ± 0.63 s, indicating a significant improvement effect. The CG athletes’ average time decreased from about 2.85 ± 0.35 to 2.83 ± 0.46 s, and the improvement effect was not significant.

### 3.4. Baseline Movement Stroke Effect Test Results

The research examined the effectiveness of the baseline movement stroke. First, the number of hits was tested. Then, the athlete’s ability to complete the baseline forehand movement, the baseline backhand movement, and the baseline one forehand and one backhand movement within 1 min was tested. All testing methods was prevalidated, and their intragroup correlation coefficients were greater than 0.85. The standard errors (SEM) of the tests were 0.5 forehand strokes, 0.6 backhand strokes, and 0.4 combined forehand and backhand strokes, ensuring the reliability of the data. The results are shown in Table 4.

**Table 4:** Test results of the number of strokes before and after training for both groups of athletes.

Batting style	Control group	Experimental group	*t*‐Values	*p*‐Values
**Before training**				

Forehand movement hitting (times)	16.31 ± 0.84	16.21 ± 0.21	−0.63	0.62
Backhand movement stroke (times)	14.51 ± 0.76	14.42 ± 0.63	0.36	0.48
One forehand and one backhand movement stroke (times)	10.30 ± 0.38	10.42 ± 0.56	−0.85	0.63

**After training**				

Forehand movement hitting (times)	16.89 ± 0.76	17.96 ± 0.67	3.21	0.02
Backhand movement stroke (times)	15.01 ± 0.35	16.42 ± 0.48	4.63	0.03
One forehand and one backhand movement stroke (times)	10.96 ± 0.47	12.11 ± 0.32	4.35	0.01

In Table 4, the shooting down times of the three hitting methods of the EG athletes before training were 16.21 ± 0.21, 14.42 ± 0.63, and 10.42 ± 0.56, respectively. The shooting down times of the CG athletes using the three hitting methods were 16.31 ± 0.84, 14.51 ± 0.76, and 10.30 ± 0.38, respectively, and *p* > 0.05. After their respective training, the shooting frequency of the EG athletes before training was 17.96 ± 0.67, 16.42 ± 0.48, and 12.11 ± 0.32, respectively. The shooting down times of the three hitting styles of the CG athletes were 16.89 ± 0.76, 15.01 ± 0.35, and 10.96 ± 0.47, respectively, and *p* < 0.05. The improvement range of EG athletes in the three hitting styles was about 1.75 times, 2.00 times, and 1.69 times, respectively. All of them were far greater than the minimum true change value of the test. This indicated that the improvement was real and meaningful, rather than caused by measurement errors.

As shown in Table 5, before training, the average depth of impact score of the EG athletes was 30.61 ± 1.42 points. The average depth of hitting score of the CG athletes was 30.25 ± 1.55 points. After *t*‐test, there was no SSD in the number of hits between the two groups of athletes before training (*t* = −0.34, *p* = 0.85). However, after training, the average depth of hitting score of the EG athletes significantly increased to 31.98 ± 1.98 points. The average depth of hitting score of the CG athletes was only 30.56 ± 1.94 points. The average depth of impact improvement for EG and CG athletes was approximately 1.37 and 0.31, respectively. At this point, after *t*‐test, the difference in hitting depth scores between the two groups of athletes after training was statistically significant (*t* = 6.42, *p* = 0.02). This indicated that the EG of athletes had a more significant improvement in the depth of stroke score after training. Further analyzing the changes of *t*‐value and *p*‐value before and after training of the two groups of athletes, the *t*‐value of the EG of athletes before and after training was 6.35, and the *p*‐value was 0.03. This indicated that the EG of athletes had a significant improvement in the depth of stroke score after training. While the *t*‐value of the CG athletes before and after training was −0.06 and the *p*‐value was 0.45. This indicated that there was no SSD in the depth of stroke score of the CG athletes before and after training. The results of the hitting accuracy test are shown in Figure 8.

Figure 8Comparison of the results of batting accuracy before and after training of the two groups of athletes. (a) Experimental group. (b) Control group.

**Figure:**
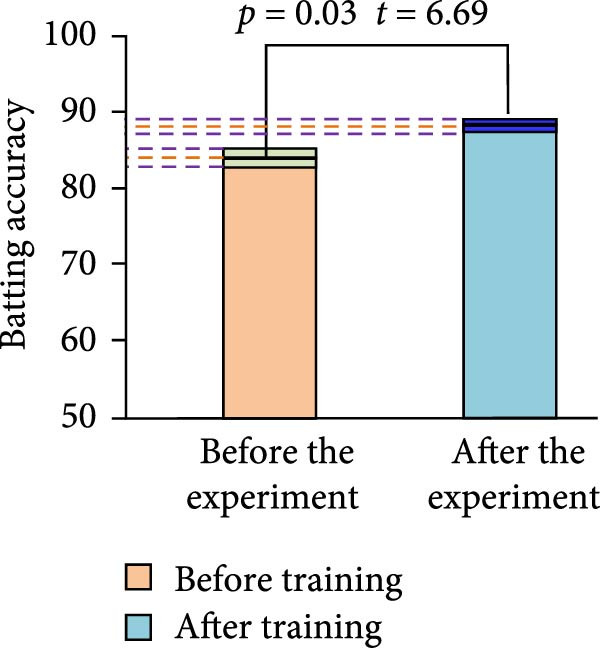
(a)

**Figure:**
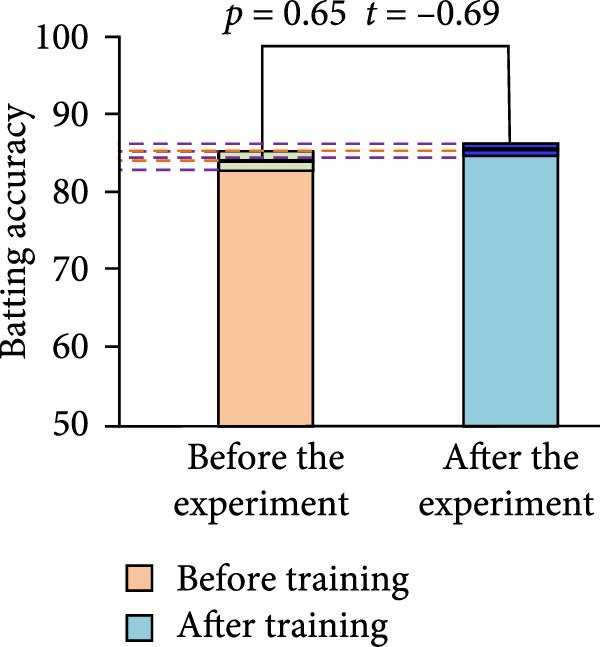
(b)

**Table 5:** Results of depth of stroke test scores before and after training for both groups of athletes.

Time	Experimental group	Control group	*t*‐Values	*p*‐Values
Before training	30.61 ± 1.42	30.25 ± 1.55	−0.34	0.85
After training	31.98 ± 1.98	30.56 ± 1.94	6.42	0.02
*t*‐Values	6.35	−0.06	—	
*p*‐Values	0.03	0.45		

In Figure 8, before training, the mean value of stroke accuracy of the EG of athletes was 83.21% ± 1.42%. The mean value of depth of stroke score of the athletes in the CG was 82.11% ± 2.42%. However, after the training, the mean value of stroke accuracy of the athletes in the EG significantly increased to 88.12% ± 1.96%. However, the mean value of stroke accuracy of the athletes in the CG was only 84.12% ± 1.96%. The hitting accuracy of EG athletes and CG athletes increased by approximately 4.91 and 2.01, respectively. At this time, after the *t*‐test, there was a SSD between the depth of stroke scores of the two groups of athletes after training. This indicated that the EG of athletes had a more significant improvement in hitting accuracy after training.

### 3.5. Post Hoc Efficacy Analysis of Main Exercise Indicators

The posttreatment efficacy analysis of key exercise indicators is shown in Table 6. According to Table 6, there were differences in the robustness of the research results. For the large effect indicators (FMS total score, standing long jump, fan‐shaped run, number of hits, and accuracy of hits), the statistical power was higher than 0.99. This indicated that the current sample size was sufficient to detect significant differences in these indicators, and the results are very robust. However, for small effect sizes, such as forehand and backhand lateral movements, the statistical power was only 0.09–0.12. This was far below the standard of 0.8 and indicated that the sample size was insufficient to reliably detect the true effects of these indicators. There was also a high risk of Type II errors. Although the hitting depth score reached the equivalent strain, its statistical power was 0.65, which was still lower than the ideal level. These findings suggested that future research required larger sample sizes to ensure sufficient detection power for all types of indicators, especially for those with relatively small effect sizes in sports performance indicators. Furthermore, upon analyzing the existing data, it was found that the expected group × time interaction of all indicators is significant. This indicated that the improvement in all indicators of EG trained with PFT was significantly greater than that of CG trained with conventional training.

**Table 6:** Post hoc efficacy analysis of key indicators.

Test metrics	Comparison type	Calculation value of effect quantity (*d*/*d*_*z*_)	Effect size	Statistical efficacy (power)	Stability evaluation of results	Group × time interaction recommendation results
FMS total score	Within group	1.85	Big	>0.99	The efficacy is extremely sufficient, and the results are very robust.	Significant
Standing long jump	Intergroup	0.19	Big	>0.99	The efficacy is extremely sufficient, and the results are very robust.	Significant
Fan run	Intergroup	5.07	Big	>0.99	The efficacy is extremely sufficient, and the results are very robust.	Significant
Forehand lateral movement	Intergroup	−0.11	Small	0.09	Insufficient efficacy and low robustness of results.	Significant
Backhand lateral movement	Intergroup	−0.18	Small	0.11	Insufficient efficacy and low robustness of results.	Significant
The number of times the forehand moves and hits the ball	Intergroup	1.49	Big	>0.99	The efficacy is extremely sufficient, and the results are very robust.	Significant
Number of backhand movements hitting the ball	Intergroup	3.36	Big	>0.99	The efficacy is extremely sufficient, and the results are very robust.	Significant
A swing with alternating forehand and backhand strokes	Intergroup	2.86	Big	>0.99	The efficacy is extremely sufficient, and the results are very robust.	Significant
Hit depth score	Intergroup	0.72	Middle	0.65	Insufficient efficacy, moderate robustness of results.	Significant
Batting accuracy	Intergroup	2.04	Big	>0.99	The efficacy is extremely sufficient, and the results are very robust.	Significant

## 4. Discussion

### 4.1. PFT Improves Athletes’ FMS Test Scores

FMS test was a kind of test to assess athletes’ overall movement control stability, body balance, flexibility, proprioception, and other abilities [24]. The research results showed that after 12 weeks of PFT intervention, the total FMS score of the EG athletes significantly increased from 14.2 ± 1.8 points before training to 17.8 ± 1.5 points. Numerous studies have shown that a total FMS score of 14 or less was a key threshold for predicting sports injury risk in athletes. Individuals with scores below this threshold were significantly more likely to be injured. After training, the athlete’s FMS total score exceeded the recognized injury risk threshold. Second, the increase in scores reflected functional improvements directly related to tennis performance. A significant improvement in dominant lower‐limb movements, such as squats, hurdle steps, and straight‐line lunges, indicated better joint alignment, stability, and power transmission efficiency in bottom‐line movements, such as kicking, emergency stops, changing directions, and leg splits. The improvement in shoulder flexibility score indicated that athletes were able to perform shoulder flexion, abduction, and other movements more smoothly in serving, high‐pressure shots, and large forehand and backhand strokes. The enhancement of trunk stability and rotational stability ensured that power could be efficiently transmitted from the lower limbs to the upper limbs and racket through a stable core during open and closed stance shots, improving hitting power and accuracy. Overall, the increase in FMS test scores indicated a significant improvement in the coordination and stability of the athlete’s body, which further enhanced their ability to hit the ball from the baseline. This was consistent with the findings of Fitton Davies et al. [25] explored the relationship between FMS composite scores (FMS‐CCs) and athletic performance among adolescents. It was found that children and adolescents with high FMS scores had better performance in agility, running speed, strength, and cardiovascular endurance [25]. Pollen et al. [26] explored whether FMS‐CCs differed between high school, college, and professional athlete groups. All of these results indicated that the FMS‐CC was related to the athletes’ sport level. Sports with high FMS‐CCs could better improve athletes’ performance.

### 4.2. PFT Improves Athletes’ Fitness Faster

PFT prevented sports injuries and improves athletic coordination by developing deep small muscle strength. This kind of training ensured the growth of strength while taking into account the overall balance and coordination, which was useful for the development of athletes’ strength, speed, endurance, and agility. In the 100‐m sprint event, the average EG time of athletes after training decreased from 11.24 ± 0.21 to 10.99 ± 0.21, with *p* < 0.05. The increase in speed means that athletes had a higher peak speed when dealing with short balls, rushing towards the net, or chasing extremely open angle balls. In the standing long jump event, the average EG time of athletes after training increased from 2.51 ± 0.23 to 2.60 ± 0.23, with *p* < 0.05. A significant improvement in standing long jump performance during baseline hitting in tennis means athletes could better “penetrate” the ground. This allowed them to generate greater force and transmit it to their arms and racket through their core muscle group. This directly contributed to improved hitting depth and speed. After training, the EG fan‐shaped running performance of athletes increased from 16.61 ± 0.23 to 17.69 ± 0.23, with *p* < 0.05. Fan shaped running ability means that athletes could react faster to large angle shots and efficiently move to the optimal hitting point, preparing for a full shot.

The above results indicated that PFT was more prominent in enhancing the athletes’ physical performance, which could lay the foundation for the enhancement of the athletes’ physical reserves. This type of training not only improved the athletes’ neural reaction speed ability, but also significantly improved the athletes’ vertical jump ability by optimizing the movement patterns, such as deep squatting. This agreed with the findings of Wang et al. [27] and Bashir et al. [28]. Using a meta‐analysis, Wang et al. [27] investigated how high‐intensity FT affected athletes’ fitness and performance in particular sports. High‐intensity FT improved upper and lower extremity muscle strength, explosiveness, flexibility, and sport‐specific performance with small to substantial effect sizes, according to the results [27]. Bashir et al. [28] investigated how FT affected athletes’ functional movements, jumping, and sprinting. According to the findings, FT interventions enhanced athletes’ performance [28].

### 4.3. PFT Improves the Ability to Perform Basic Movement Strokes

After 12 weeks of PFT, the performance of the EG of athletes in the baseline movement stroke was significantly improved. Not only the number of strokes increased significantly, but also the depth and accuracy of the strokes. In terms of the number of hits, the EG showed an improvement of 1.75 times, 2.00 times, and 1.69 times in the three types of hitting methods (forehand movement hitting, backhand movement hitting, and alternating forehand and backhand movement hitting). These values were far greater than the minimum true change values in each test. This result indicated that the improvement in hitting frequency of the EG athletes was not caused by measurement errors, but by the real ability enhancement brought about by training. Second, in terms of hitting depth, the EG’s score increased from 30.61 ± 1.42 before training to 31.98 ± 1.98 after training, with an improvement of 1.37 points, while the CG only increased by 0.31 points. The depth of hitting was an important indicator of hitting quality. Improvement in this area not only showed that athletes progressed in strength control and technical stability, but also reflected the overall positive effect of training on hitting performance. In terms of hitting accuracy, the EG’s accuracy increased from 83.21% to 88.12%, with a growth rate of 4.91%, while the CG’s growth rate was only 2.01%. This result indicated that the EG could significantly improve the landing control ability of the ball while maintaining the frequency and depth of hitting.

In conclusion, PFT could significantly improve athletes’ baseline movement stroke ability, including the number, depth, and accuracy of strokes, thus, optimizing the overall quality of baseline movement stroke. This was due to the fact that upper‐body training in PFT promoted coordination between the muscles. This enabled the athletes to adjust their body position faster and to be more effective in delivering power during the baseline movement stroke. The stretching and warm‐up component of the training improved flexibility and suppleness, which allowed the athletes to change direction faster and adjust their stroke position more effectively during baseline movement. In addition, PFT focused on optimizing athletes’ movement patterns by using specific training movements (e.g., one‐knee kneeling on a balance board with a solid ball thrown sideways, etc.) to improve athletes’ stability and accuracy in complex movements. At the same time, the training also enhanced the athletes’ motion perception. This enabled them to more accurately determine the landing point and speed of the ball, and thus, make more precise striking movements. Xiao et al. [29] conducted an investigation on the effects of FT on athletes’ physical performance. According to the findings, FT significantly improved athletes’ power, speed, agility, balance, and muscle strength [29]. This indicated that PFT could improve flexibility and balance by optimizing movement patterns, enhancing muscle strength and coordination [30].

### 4.4. Evaluation and Discussion of Routine Training in the CG and PFT Training in the EG

In the study, the CG followed the conventional tennis physical training plan, which included foam axis rolling, static stretching, traditional strength training (such as dumbbell over press and barbell squat), and basic core stability exercises (such as flat support and hip bridge). This type of training mode was widely used in amateur and professional sports teams, focusing on general strength, endurance, and flexibility development. However, compared to the PFT of the EG, conventional training lacked a targeted design for tennis‐specific movement patterns, multijoint coordination, neuromuscular control, and dynamic stability in multiple planes. For example, the CG’s training content did not specifically simulate spinning chains, emergency stops, changing directions, or dynamic balance challenges in tennis hitting. These were precisely the types of exercises emphasized by PFT, such as single‐leg stability, medicine ball throwing, and spinning core training. Therefore, although both groups of athletes received the same duration and frequency of structured training, the PFT group showed more significant improvements in FMS, specialized physical fitness (e.g., fan‐shaped running and jumping), and hitting performance indicators. These results highlighted the superiority of PFT as a highly targeted training method for optimizing power chain efficiency and enhancing specialized movement control and quality. This result was consistent with the current emphasis in sports training on the concept that “action quality is superior to isolated muscle strength.” This indicated that, in the future, tennis ball energy plans should incorporate more FT content that was closely integrated with specialized techniques and biomechanical mechanisms, beyond traditional methods.

## 5. Conclusion

The study focused on 32 national second level and above college tennis players aged 20–22 years old. After a 12 week, three‐time, 1‐h intervention, only significant improvements were observed in the total FMS score and specialized physical fitness indicators such as standing long jump and fan‐shaped running in the PFT group. These changes were consistent with the multijoint coordination and power transmission required for tennis baseline initiation, emergency stops, and stride shots. In terms of bottom line hitting, the PFT group’s improvement in hitting frequency, depth, and accuracy was greater than the minimum true change value, indicating reliability. The traditional training used by the CG maintained basic physical fitness. However, it did not show similar benefits due to the lack of design for tennis power chain and dynamic stability. The results were only applicable to college tennis populations with similar conditions to this study. They could not be extrapolated to teenagers or professional players. This was due to the high homogeneity of sample size, gender, age, and technical level.

Although the research results indicate that PFT has a positive impact on tennis players, there are limitations. (1) The study lacks follow‐up testing on training effectiveness and conducts a posttest immediately after 12 weeks of training. However, it can not determine how long the observed positive effects could be sustained. Subsequently, a retention test will be conducted 4, 8, or 12 weeks after the cessation of the training intervention to evaluate the persistence and stability of the PFT effect. (2) The samples for the study are all from tennis teams of the same sports school. While this setting is useful for managing the training environment, coaching level, and testing process, it significantly restricts the external validity and generalizability of the research results. In the future, multicenter and large sample studies are needed to verify the effectiveness of PFT in different populations.

## Ethics Statement

Ethics Committee approval was obtained from the Institutional Ethics Committee of “School of Physical Education, Hebei Normal University, Hebei, Shijiazhuang” to the commencement of the study. Confirmation that informed consent was obtained from the study participants, includes information regarding informed consent obtained from the study participant’s parent or legal guardian for any participant below the age of consent. Confirmation that the guidelines outlined in the Declaration of Helsinki were followed.

## Disclosure

All authors have read the manuscript and that the content of this manuscript or a major portion thereof has not been published in any other refereed journal and it is not being submitted fully or partially for publication elsewhere.

## Conflicts of Interest

The authors declare no conflicts of interest.

## Funding

No funding was received for this research.

## Data Availability

The data that support the findings of this study are available from the corresponding author upon reasonable request.
